# Perovskite-Inspired Lead-Free Ag_2_BiI_5_ for Self-Powered NIR-Blind Visible Light Photodetection

**DOI:** 10.1007/s40820-020-0371-0

**Published:** 2020-01-20

**Authors:** Vincenzo Pecunia, Yue Yuan, Jing Zhao, Kai Xia, Yan Wang, Steffen Duhm, Luis Portilla, Fengzhu Li

**Affiliations:** Institute of Functional Nano & Soft Materials (FUNSOM), Jiangsu Key Laboratory for Carbon-Based Functional Materials and Devices, Soochow University, 199 Ren’ai Road, Suzhou, 215123 Jiangsu People’s Republic of China

**Keywords:** Perovskite-inspired semiconductor, Silver bismuth iodide, Self-powered photodetector, NIR-blind photodetector, Visible light photodetector

## Abstract

In recent years, solution-processible semiconductors with perovskite or perovskite-inspired structures have been extensively investigated for optoelectronic applications. In particular, silver–bismuth–halides have been identified as especially promising because of their bulk properties and lack of heavily toxic elements. This study investigates the potential of Ag_2_BiI_5_ for near-infrared (NIR)-blind visible light photodetection, which is critical to emerging applications (e.g., wearable optoelectronics and the Internet of Things). Self-powered photodetectors were realized and provided a near-constant ≈ 100 mA W^−1^ responsivity through the visible, a NIR rejection ratio of > 250, a long-wavelength responsivity onset matching standard colorimetric functions, and a linear photoresponse of > 5 orders of magnitude. The optoelectronic characterization of Ag_2_BiI_5_ photodetectors additionally revealed consistency with one-center models and the role of the carrier collection distance in self-powered mode. This study provides a positive outlook of Ag_2_BiI_5_ toward emerging applications on low-cost and low-power NIR-blind visible light photodetector.

**Electronic supplementary material:**

The online version of this article (10.1007/s40820-020-0371-0) contains supplementary material, which is available to authorized users.

## Highlights

- The photodetection capabilities of emerging perovskite-inspired lead-free Ag_2_BiI_5_ are investigated.
- In self-powered mode, a near-constant photoresponse through the visible with a NIR rejection ratio of > 250 is obtained.
- Optoelectronic characterization provides insight into the interplay among efficiency, collection distance, and film micro-/nano-structure.

**Electronic supplementary material:**

The online version of this article (10.1007/s40820-020-0371-0) contains supplementary material, which is available to authorized users.

## Graphical abstract

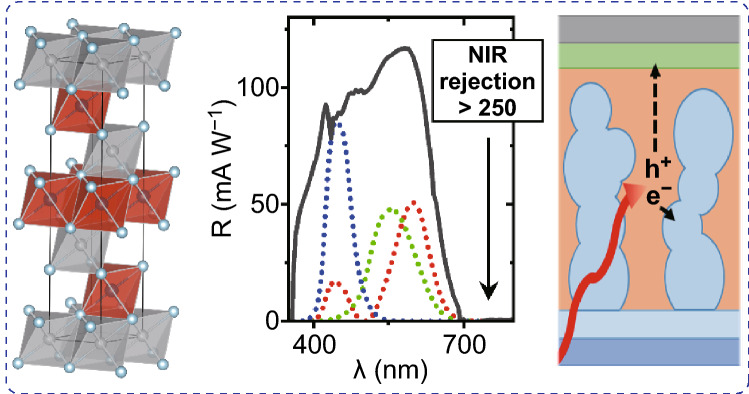

## Introduction

Visible light photodetection is central to many emerging optoelectronic applications (e.g., wearable optoelectronics [1], lab-on-chip and biomedicine [2, 3], and the Internet of Things (IoT) [4]). Emerging solution-processed semiconductors such as perovskite or perovskite-inspired semiconductors [5–9] are highly attractive to this end, as they are compatible with unconventional substrates and facile, low-cost manufacturing. Therefore, they can potentially lead to visible light photodetectors with unique form factors that could easily be implemented in the objects and environments of daily life. Semiconductors that have potential for visible light photodetection should feature an absorption spectrum that precisely covers the visible range, as this will avoid the use of near-infrared (NIR) cut filters, which are detrimental to photoconversion efficiency, complexity, and cost [10]. In addition, an in-band absorption coefficient greater than 1 × 10^5^ cm^−1^ is highly desirable because it would enable visible light photodetectors to have active layer thicknesses in the hundreds of nanometers (e.g., potentially leading to high robustness against optical spatial crosstalk [11] at reduced pixel sizes within an imaging device [12, 13]). Moreover, the ability to work in self-powered mode (i.e., with 0 V as the applied bias) is highly desirable, as it would ensure compatibility with remote sensing and off-the-grid applications. Finally, semiconductors made of non-toxic or low-toxicity elements are most attractive, as they could easily be used in the many environments required by emerging application domains.

Among perovskite-inspired solution-processed semiconductors, rudorffites have attracted increasing attention over the last few years particularly because of their three-dimensional structure and because they do not contain heavily toxic elements [14–24]. Their structure relies on metal-halide [MX_6_] octahedra (where M is a monovalent or trivalent metal, e.g., Bi, Sb, Cu, Ag, and X is a halide) as the building blocks of a three-dimensional lattice featuring an edge-sharing packing motif [17–19]. In particular, silver–bismuth iodides (Ag_*a*_Bi_*b*_I_*x*_, *x* = *a* + 3*b*) have been identified as particularly promising, as evidenced by their increasing attention in photovoltaic research [14, 15, 17–24]. In fact, their potential is confirmed by a rapid rise in solar power conversion efficiency (up to 4.3%) [14, 15, 19–23]. Despite their promising optoelectronic properties, the capabilities of silver–bismuth iodides for photodetection have not been explored to date.

In order to assess the potential of silver–bismuth iodides for visible light photodetection, this study explores Ag_2_BiI_5_ (which is a prominent embodiment [19, 23]), with a focus on how its photodetector performance relates to processing strategies and structural/optoelectronic properties. It was found that Ag_2_BiI_5_ photodetectors can deliver a spectrally uniform response through the visible range while simultaneously achieving large NIR rejection and maintaining a linear behavior over more than 5 orders of magnitude of incident optical power. In addition, by investigating the static and transient photodetector response, we gain physical insight into Ag_2_BiI_5_ optoelectronics, which allows us to identify the present performance limitations and the potential strategies for further improvement. Therefore, this study represents a major step in the development of NIR-blind visible light lead-free perovskite-inspired photodetectors for emerging applications, including wearable optoelectronics and IoT.

## Experimental

### Materials

All chemical reagents were used as received and without further purification. BiI_3_ (99.998%, Sigma-Aldrich); AgI (99.999%, Sigma-Aldrich); titania paste (18NR-T, Greatcell Solar); titanium isopropoxide (TTIP) (≥ 97.0%, Sigma-Aldrich); 2,2′,7,7′-tetrakis[*N*,*N*-di(4-methoxyphenyl)amino]-9,9′-spirobifluorene (Spiro-OMeTAD) (> 99.5%, Lumtec); hydriodic acid (HI) (57 wt% in H_2_O, 99.95%); hydrochloric acid (HCl) (AR, 12.06 mM, Enox.); dimethylsulfoxide (DMSO) (> 99.7%, Acros); dimethylformamide (DMF) (> 99.7%, Acros); ethanol (AR, YongHua); chlorobenzene (CB) (> 99%, J&K); chloroform (CF) (> 99.7%, YongHua); anhydrous isopropyl alcohol (an.IPA) (99.5%, Sigma-Aldrich); and Ag slugs (99.999%, Alfa Aesar).

### Photodetector Fabrication

Glass substrates with patterned fluorine-doped tin-oxide (FTO) electrodes (Ying Kou You Xuan Trade Co., Ltd) were cleaned in an ultrasonic bath (sequentially in acetone, isopropyl alcohol, and ultrapure water) and then treated with UV-ozone for 15 min. A TiO_2_ layer was then deposited, either in the form of a compact film (c-TiO_2_) or as a mesoporous layer (mp-TiO_2_) on top of a compact film (as needed). After a Ag_2_BiI_5_ layer was deposited, the photodetector stack was completed using Spiro-OMeTAD and Ag. Details on the deposition conditions of each of the layers are as follows.

#### c-TiO_2_

380 μL of TTIP was added dropwise into 2.53 mL of an.IPA while being stirred; 35 μL of HCl (2 mM in H_2_O) and 2.53 mL of an.IPA were mixed in a separate vial; TTIP:an.IPA was added dropwise into HCl:an.IPA, and the resulting solution was stirred at room temperature overnight; the solution was spin-coated at 4000 rpm (40 s); the films were finally annealed at 490 °C (30 min).

#### mp-TiO_2_

The titania paste was mixed with ethanol (2:7); the mixture was spin-coated (7000 rpm, 60 s); and the layers were finally annealed (450 °C, 30 min).

#### Ag_2_BiI_5_ (HIA)

Powders of AgI and BiI_3_ (molar ratio = 2:1) were dissolved in HI:DMSO:DMF = 0.2:3:1 (0.3 M); the solution was spin-coated (3000 rpm, 20 s); and the layers were finally annealed (130 °C, 15 min).

#### Ag_2_BiI_5_ (ASP)

Powders of AgI and BiI_3_ (molar ratio = 2:1) were dissolved in DMSO:DMF = 1:3 (0.3 M); the solution was spin-coated (3000 rpm, 30 s); 20 s into the spin-coating cycle, CF (250 μL) was dispensed onto the substrate; subsequently, samples were annealed at 100 °C (for several seconds until the Ag_2_BiI_5_ film changed color); then their temperature was raised to 190 °C (over 10 min) and finally kept at 190 °C (for 10 min).

#### Ag_2_BiI_5_ (HC)

Powders of AgI and BiI_3_ (molar ratio = 2:1) were dissolved in DMSO:DMF = 1:3 (0.3 M); substrates and solutions were preheated at 100 °C; the hot solution was spin-coated on a preheated substrate (8000 rpm, 30 s); annealing followed (190 °C, 10 min).

#### Spiro-OMeTAD

A solution was prepared in CB (74 g L^−1^) and was spin-coated at 3000 rpm (30 s).

#### Ag Cathodes

100-nm-thick silver films were thermally evaporated in high vacuum through a shadow mask, leading to a photodetector active area of 7.25 mm^2^.

### Characterization

Scanning electron microscopy (SEM) images were acquired through a Carl Zeiss Supra 55 system. UV–Vis absorption spectra were acquired using a SPECORD S 600 spectrometer (Analytik Jena). X-ray diffraction (XRD) spectra were acquired using a D8 ADVANCE X-ray diffractometer (Bruker). UPS spectra were measured using an Ultra DLD X-ray/ultraviolet photoelectron spectrometer (Kratos). External quantum efficiency (EQE) spectra were acquired (in air) with a home-built setup relying on a monochromated light source (Zolix, Omni-λ2005i), a calibrated power meter assembly (Thorlabs PM200 and Thorlabs S120VC), and a source meter (Keithley 6420). Steady-state photocurrent-power measurements were conducted (in air) with a home-built setup relying on a high-power 525-nm LED source (SOLIS-525C), optical filters (Zolix), and a semiconductor parameter analyzer meter (Tektronix, 4200A-SCS). Transient photocurrent measurements were conducted (in air) with a home-built setup: Photodetectors were illuminated with an LED (Osram LV CK7P) emitting at 505 nm and driven by a custom-made power amplifier controlled by a signal generator (Pico Technology, PicoScope 5444B); the photodetectors were biased at 0 V, and their current was fed to a variable-gain current amplifier (FEMTO, DHPCA-100). The amplifier output was then acquired using an oscilloscope (Pico Technology, PicoScope 5444B). The Hall effect characterization was conducted using a Lakeshore 8404 (at room temperature, in air, in the dark, and with an a.c. magnetic field of 0.42291 T at 100 mHz) on 1 × 1 cm^2^ van der Pauw samples (spin-coated silver–bismuth iodide on glass).

## Results and Discussion

**Fig. 1:**
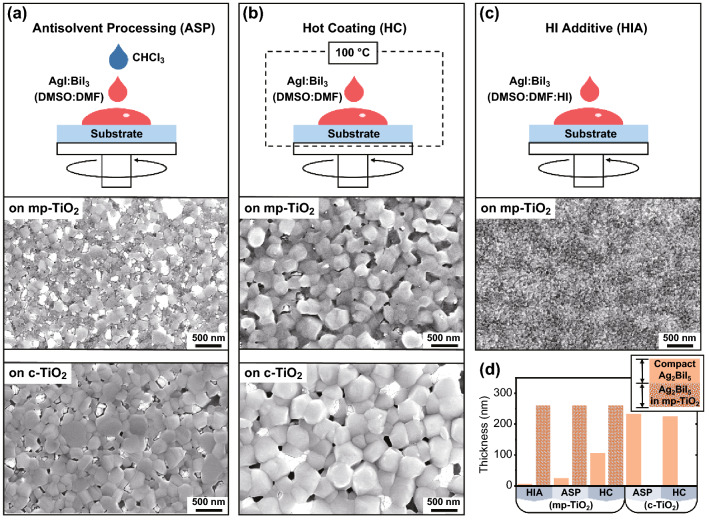
Ag_2_BiI_5_ layers deposited through **a** ASP, **b** HC, and **c** HIA. For each, a schematic of the deposition process is shown as well as SEM images of the resulting layers on different substrates. **d** Histogram showing the average thickness and composition of the Ag_2_BiI_5_ layers, as determined through cross-sectional SEM (Fig. S5)

For a comprehensive assessment of the potential of Ag_2_BiI_5_ for photodetection, its deposition was conducted using a range of solution-based methods, which resulted in polycrystalline layers with different microstructures, crystallographic properties, and phase purities. These methods (schematically presented in Fig. 1) all relied on a base solution of AgI and BiI_3_ precursors in DMF:DMSO and a spin coating cycle followed by an annealing step (see the Experimental section for details). In brief, the first of these methods involves antisolvent processing (ASP): when the base precursor solution is spin-coated, droplets of an antisolvent are dispensed onto the spinning substrate to condition the Ag_2_BiI_5_ crystallization process. The second deposition method consists of a hot coating (HC) approach, which involves the spin coating of a preheated solution on a preheated substrate. This was explored with the aim of thermally conditioning the film microstructure and grain size. As a final deposition method (HIA in short form), we added a tiny amount of hydroiodic acid (HI(*aq*)) to the base solution as a means of increasing the precursor solubility. The aim here was to overcome the challenge posed by the limited solubility of AgI [19]. Further details on the deposition methods can be found in the Experimental section and in Figs. S1–S3.

Suitable optimization of antisolvent processing and hot coating enabled the deposition of compact Ag_2_BiI_5_ layers both on an untextured substrate (coated with a compact TiO_2_ film; c-TiO_2_ in short form) and on a textured substrate (firstly coated with a compact TiO_2_ film and subsequently with a mesoporous TiO_2_ layer; mp-TiO_2_ in short form). TiO_2_ was used as substrate coating (before Ag_2_BiI_5_ deposition), because it can act as an electron transport layer within a photodetector stack. Both ASP and HC resulted in polycrystalline films of variable grain size, namely as large as ≈ 500 nm in lateral dimensions on the untextured substrates but generally smaller (≈ 10–300 nm) on the textured counterparts (Fig. 1a, b). However, these Ag_2_BiI_5_ layers also featured domains of an impurity phase (i.e., the bright features in the SEM images in Fig. 1a, b). As this impurity phase is likely related with the precipitation of AgI, the HIA approach was pursued. Indeed, this method successfully achieved an increase in precursor solubility. (At room temperature, clear solutions were obtained up to a concentration of 0.8 M.) We found, however, that the HIA approach did not deliver compact Ag_2_BiI_5_ films on an untextured substrate. This was verified over a broad space of processing parameters (involving variations in solution concentration, amount of solution dispensed on the substrate, spin coating speed, and spin coating cycle structure) (Fig. S4). This could be due to the reduction in the supersaturation level attained with HI(*aq*) addition in the precursor solution, leading to a dramatic increase in the grain growth rate over the nucleation rate. For the purpose of providing a high density of nucleation sites and thus helping the formation of an Ag_2_BiI_5_ layer, we investigated the application of the HIA approach on substrates pre-coated with a mesoporous TiO_2_ layer. In this case, an Ag_2_BiI_5_ layer was successfully formed and its fine grains were predominantly interspersed within the mesoporous TiO_2_ matrix (Fig. 1c). In contrast to our findings with antisolvent processing and hot coating, no obvious impurity phase was observed through SEM imaging in HIA samples. Apart from surface morphology, it is important to note that the different deposition methods and substrate texturing resulted in Ag_2_BiI_5_ layers with different cross-sectional structures. On mesoporous-TiO_2_-coated substrates, the photoactive layer consisted of two regions (see the cross-sectional SEM images in Fig. S5): a ≈ 250 nm thick region embedded in the TiO_2_ matrix regardless of the processing method; a region that overlay the TiO_2_ matrix, with a thickness of ≈ 5 nm in HIA samples, ≈ 30 nm in the APC case, and ≈ 100 nm in the HC samples (Fig. 1d). By contrast, the APC and HC samples on untextured substrates featured only a compact Ag_2_BiI_5_ layer, and the thickness of which was in the range of 200 nm in both cases (Fig. 1d).

**Fig. 2:**
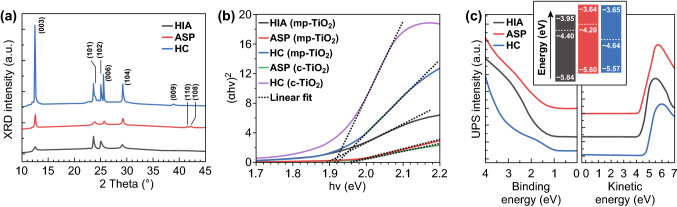
**a** XRD patterns, **b** Tauc plots, and **c** UPS intensity (valence band states (left) and He I secondary electron onset (right)) from Ag_2_BiI_5_ layers fabricated using the different processing methods detailed in the text. Inset of **c**: energy levels of the layers deposited through the different methods

The XRD patterns confirmed the structural identity as Ag_2_BiI_5_ of the different Ag_2_BiI_5_ layers (Fig. 2a). A trigonal crystal structure ($R\bar{3}m$) was observed in all cases, with the positions of the XRD peaks being in agreement with the literature [20]. In addition, all films presented a direct optical gap *E*_g_ of approximately 1.9 eV (*λ*_onset_ ≈ 650 nm), as derived from the Tauc plots (Fig. 2b). Finally, the in-band absorption coefficient was > 1 × 10^5^ cm^−1^ (Fig. S6), closely matching the literature data [19, 20, 22]. This confirmed the potential of any of these Ag_2_BiI_5_ layers for visible light photodetection.

With the aim of determining the key energy levels relevant to photodetector operation, we conducted ultraviolet photoelectron spectroscopy (UPS) on the different types of Ag_2_BiI_5_ layers (Fig. 2c). The derived valence band maxima $E_{\text{v}}$ were at ≈ 5.6–5.8 eV below the vacuum level. Considering their optical gaps, we estimate that their conduction band minima *E*_c_ were at 3.65–3.95 eV below the vacuum level, under the assumption that excitonic effects were negligible. These band edge energies were in reasonably good agreement with the literature [22, 23]. In addition, the work functions *W*_F_ were equal to 4.40 eV (HIA), 4.20 eV (ASP), and 4.64 eV (HC). The *W*_F_ difference among samples deposited using different methods suggested that the number of defects acting as dopants was affected by the details of the deposition process.

**Fig. 3:**
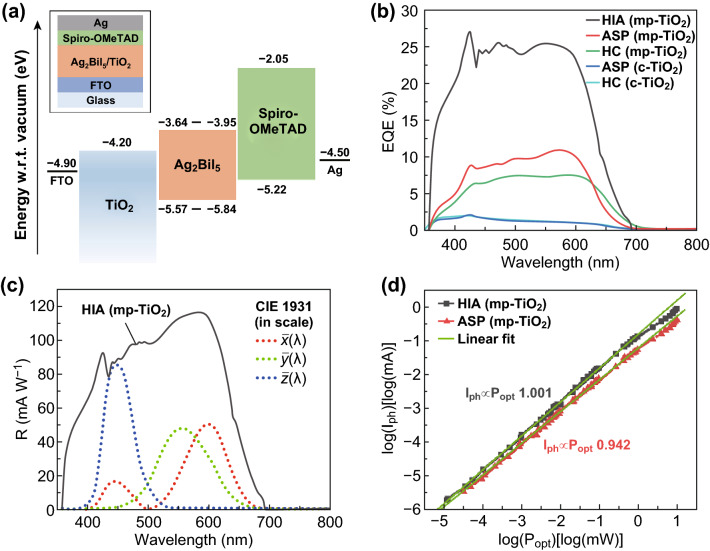
**a** Frontier energy levels of all layers within the photodetector stack (inset: device stack). **b** EQE versus wavelength of photodetectors realized with different processing methods and on different ETL structures. **c** Spectral responsivity of HIA (mp-TiO_2_) photodetectors. Overlaid are the CIE 1931 XYZ standard observer color matching functions (in scale). **d** Photocurrent versus optical power of HIA and ASP photodetectors along with power-law fits

To assess the photoconversion and photodetection capabilities of the different Ag_2_BiI_5_ layers, devices were fabricated by sandwiching these layers between suitable electrode/charge-transport-layer assemblies. Fluorine-doped tin-oxide (FTO) was selected as cathode, and TiO_2_ was adopted as electron transport layer (ETL). In particular, the TiO_2_ layer consisted exclusively of a compact film (c-TiO_2_) wherever practicable or of a compact film coated with mesoporous TiO_2_ (mp-TiO_2_). As the anode plus hole transport layer (HTL), a 2,2′,7,7′-tetrakis[*N*,*N*-di(4-methoxyphenyl)amino]-9,9′-spirobifluorene (Spiro-OMeTAD)|Ag assembly was adopted. This choice of electrode and transport layer materials was derived from energy-level alignment considerations (Fig. 3a). Finally, while attempting to adopt the mainstream doping approach for Spiro-OMeTAD (addition of tert-butylpyridine (tBP) and lithium bis(trifluoromethylsulfonyl)-imide (LiTFSI)) to improve its charge transport properties, we found that tBP dissolved Ag_2_BiI_5_ (Fig. S7). Therefore, pristine Spiro-OMeTAD was employed in the final device structure.

The EQE spectra of all photodetector configurations (i.e., photodetectors incorporating HIA, ASP, and HC layers with or without mp-TiO_2_) over the wavelength range of 350–800 nm and at an applied bias of 0 V are shown in Fig. 3b. In all cases, the EQE spectra exhibited a long-wavelength onset at approximately 700 nm and featured a plateau in the range of 400–600 nm. The highest EQE of 27% was achieved with HIA devices. In addition, all photodetectors exhibited excellent spectral rejection of NIR wavelengths. This could be quantified in terms of their spectral rejection ratio (defined as ${\text{SRR}}_{\text{EQE}} \left( {\lambda_{\text{p}} ,\lambda_{\text{adj}} } \right){ \sim } = {\text{EQE}}\left( {\lambda_{\text{p}} } \right) / {\text{EQE}}\left( {\lambda_{\text{adj}} } \right)$, where $\lambda_{\text{p}}$ is a representative wavelength within the passband and $\lambda_{\text{adj}}$ is the stopband counterpart) [13]. Taking $\lambda_{\text{p}}$ within the EQE plateau, the ${\text{SRR}}_{\text{EQE}}$ of HIA photodetectors was > 250 (this represents a lower estimate in view of the limitations of our measuring apparatus) for all wavelengths $\lambda_{\text{adj}} > 750$ nm (compare with ${\text{SRR}}_{\text{EQE}} \cong 1$ for Si photodetectors over the same wavelength range). The corresponding spectral responsivity $R\left( \lambda \right)$ ($R\left( \lambda \right){ \sim } = I_{\text{ph}} \left( \lambda \right)/P_{\text{opt}} \left( \lambda \right)$, where $I_{\text{ph}}$ is the photocurrent for an incident optical power $P_{\text{opt}}$) of HIA photodetectors is shown in Fig. 3c. (See Note S1 and Fig. S8 for an estimate of the corresponding specific detectivity.) Here, $R\left( \lambda \right)$ peaks at ≈ 120 mA W^−1^ and exhibits a spectral dependence similar to that of the EQE. With respect to applications relevant to visible light photodetection, the measured $R\left( \lambda \right)$ is compared with the CIE 1931 XYZ standard observer color matching functions (Fig. 3c), which represent the ideal sensitivities for color sensing/imaging [25]. $R\left( \lambda \right)$ well encompasses all color matching functions, and its long-wavelength onset closely follows that of $\bar{x}\left( \lambda \right)$, which relates to the human sensitivity to red light. (In contrast, Si photodetectors require external filters to achieve the same.) In summary, the spectral characteristics of Ag_2_BiI_5_ (HIA) photodetectors reveal good EQE, a large spectral rejection ratio with respect to the NIR, and a close match with the long-wavelength onset of standard colorimetric functions. This establishes that Ag_2_BiI_5_ photodetectors have potential for NIR-blind visible light photodetection and, in particular, for colorimetric/color-sensing applications.

In addition to its technological implications, the EQE dataset in Fig. 3b provides critical information on the optoelectronic properties of Ag_2_BiI_5_. The broad performance range covered by the different Ag_2_BiI_5_ layers is striking, especially considering that highest performance is obtained with HIA-processed layers, which have the smallest grain size. Indeed, a general paradigm in perovskite research is that polycrystalline films with larger grain sizes achieve superior photoconversion efficiency because of a direct correlation between grain size and carrier drift length [26–29]. Having ruled out any significant impact of the differences in absorption efficiency (Note S2 and Fig. S9), we determined that the trend emerging from Fig. 3b truly reflected differences in photocarrier collection efficiency (number of collected electron–hole pairs per photogeneration event).

A lead on the discrepancy between morphology and photoconversion efficiency can be found by considering the transversal structure of the various Ag_2_BiI_5_ photoactive layers. Indeed, in addition to the fact that they feature the highest efficiency and smallest grain size, HIA layers also have the smallest proportion and thickness of a compact Ag_2_BiI_5_ region (Fig. 1d). Furthermore, intermediate thicknesses and proportions of a compact Ag_2_BiI_5_ region are found in ASP (mp-TiO_2_) and HC (mp-TiO_2_) layers, which concurrently deliver intermediate efficiency values (Fig. 1d). Finally, this same correlation extends to ASP (c-TiO_2_) and HC (c-TiO_2_) layers.

**Fig. 4:**
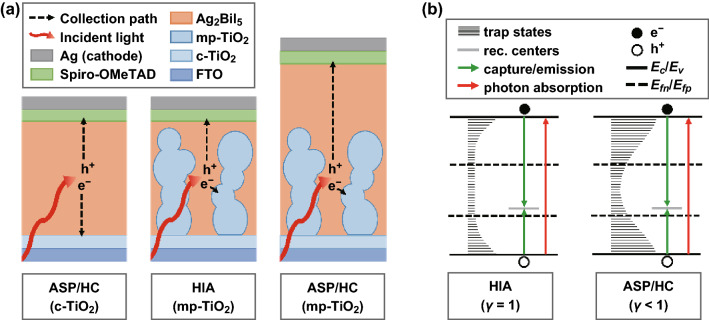
**a** Schematic of absorption and carrier collection in different Ag_2_BiI_5_ layers. **b** One-center models consistent with the optoelectronic characterization of the Ag_2_BiI_5_ layers presented in the text

Photoactive layers with a thicker region of compact Ag_2_BiI_5_ inevitably result in photocarriers having to travel a longer distance (collection distance $L$) to reach the respective electrodes (Fig. 4a). By contrast, a photoactive layer embedded within a mesoporous ETL features a particularly short collection distance for electrons (Fig. 4a). In fact, the collection distance directly affects the collection efficiency $\eta_{\text{C}}$ (to a first order, $\eta_{\text{C}} \propto 1/L$) [30]. Therefore, the observed trend in photoconversion efficiency relates to the thickness of the compact Ag_2_BiI_5_ region present in the different photoactive layers (Fig. 4a). Further evidence that the collection distance dominates the observed trend is found in the behavior of HC and ASP samples. Indeed, these samples deliver the highest performance on mp-TiO_2_ layers (i.e., the configuration that ensures an averagely shorter collection distance; see Fig. 4a). Although a quantitative analysis of the observed efficiency trend would require detailed simulations (which are beyond the scope of this work), the experimental results presented here provide insight into the optoelectronic behavior of Ag_2_BiI_5_ and the photodetector architectural requirements for self-powered operation. Indeed, the need to use a mesoporous layer for efficient photodetection directly relates to operation at a reduced electric field (as available in self-powered mode), which affects the carrier drift length (the distance travelled by the carriers prior to recombination) and the collection efficiency $\eta_{\text{C}}$.

Considering the higher efficiency achieved with devices featuring mp-TiO_2_, we could also argue that excitonic effects may play a role in the observed EQE differences. This hypothesis derives from a recent report [21] on the mismatch between the optical and electronic gaps in Ag_2_BiI_5_, notwithstanding that the role of excitons in Ag_2_BiI_5_ has not been thoroughly assessed to date. If excitonic effects were dominant in our Ag_2_BiI_5_ layers, we would have expected that having photon absorption occur primarily in the vicinity of the Ag_2_BiI_5_|TiO_2_ interface (i.e., in the region where Ag_2_BiI_5_ is interspersed with mp-TiO_2_) would aid exciton dissociation and thus deliver a higher photocurrent. This hypothesis, however, contradicts the observed EQE trends from samples processed on mp-TiO_2_. Indeed, absorption data from those samples indicate that most photons (85–90%, calculated from the relationship $1 - 10^{ - A}$, where *A* is the absorbance of the layer consisting of only Ag_2_BiI_5_ interspersed with mp-TiO_2_) are absorbed within the region in which Ag_2_BiI_5_ is interspersed with mp-TiO_2_. Therefore, similar exciton dissociation efficiencies (i.e., the number of photogenerated electron–hole pairs per photogenerated exciton) would be expected from all such samples. We thus infer that our experimental findings did not arise from excitonic effects (if any), and that differences in collection efficiencies played a dominant role in our Ag_2_BiI_5_ layers.

Further insight into the working principle of Ag_2_BiI_5_ photodetectors can be obtained by considering their photocurrent responses at variable optical power. In particular, HIA photodetectors delivered a linear response ($I_{\text{ph}} \propto P_{\text{opt}}^{\gamma }$ with $\gamma = 1.001$) over more than 5 orders of magnitude (approximately 10 nW cm^−2^ – 1 mW cm^−2^) (Fig. 3d). In the high power region ($P_{\text{opt}}$ approximately greater than 1 mW cm^−2^), the photocurrent became proportional to $P_{\text{opt}}^{0.85}$, whereas the absolute deviation from linearity was < 5 dB. By contrast, at low optical power, linearity was maintained down to the limit of our measuring apparatus. Considering the anticipated noise-determined limitation on linearity at low optical power, the upper limit on the linear dynamic range of Ag_2_BiI_5_ (HIA) was 182 dB (Note S3 and Fig. S10). Therefore, these results establish Ag_2_BiI_5_ (HIA) as a perovskite-inspired semiconductor capable of linear visible light photodetection and reveal a particularly broad linear response, suitable for many applications.

In addition to its technological significance, the observed $I_{\text{ph}} - P_{\text{opt}}$ linearity is revealing with respect to the optoelectronic properties of Ag_2_BiI_5_ (HIA). Indeed, a linear $I_{\text{ph}} - P_{\text{opt}}$ relationship constitutes a characteristic signature of operation under monomolecular recombination through a fixed number of recombination centers [31, 32]. By contrast, the $I_{\text{ph}} - P_{\text{opt}}$ relationship of HC- and ASP-deposited Ag_2_BiI_5_ is nonlinear to a variable extent, which is consistent with a power-law dependence $I_{\text{ph}} \propto P_{\text{opt}}^{\gamma }$ with $0.7 < \gamma < 0.94$ (Figs. 3d and S12). Within the framework of the Rose–Bube theory, this constitutes the typical signature of carrier trapping through an exponential distribution of defect states (Fig. 4b) [31, 32]. However, space-charge effects cannot be ruled out for devices with $\gamma \cong 0.75$ (Note S4) [33, 34]. For similar reasons, a shallow exponential tail (Fig. 4b) can be expected in the HIA case based on the sublinear $I_{\text{ph}} - P_{\text{opt}}$ trend at high power (Fig. 3d). Interestingly, the nonlinearity emerging from HC- and ASP-deposited Ag_2_BiI_5_ was accompanied by a significant sample-to-sample variability of the exponent $\gamma$. We speculate that this was due to the specifics of both processing methods. Indeed, both methods were prone to significant variability (e.g., varying thermal transients in HC and sensitivity to antisolvent release parameters in ASP), which may freeze in defect states with variable energetic depth.

**Fig. 5:**
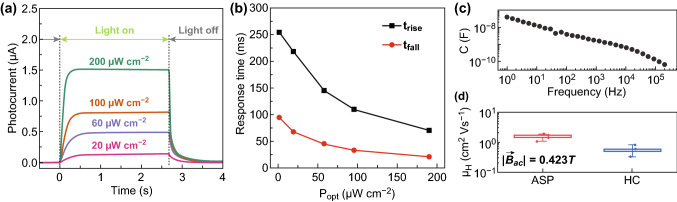
Characterization and insight into the transient response of Ag_2_BiI_5_ photodetectors. **a** Photodetector response to pulsed light (*λ* = 505 nm) of variable power. **b** Extracted rise and fall times. **c** Photodetector capacitance as a function of frequency for an applied bias of 0 V. **d** Hall mobility of Ag_2_BiI_5_ thin films

The transient photocurrent response of Ag_2_BiI_5_ (HIA) provided a valuable means of testing our mechanistic hypothesis derived from $I_{\text{ph}} - P_{\text{opt}}$ data. (Considering the suboptimal behavior of ASP- and HC-deposited Ag_2_BiI_5_, we narrow the focus in the following discussion to HIA-deposited Ag_2_BiI_5_, unless otherwise noted.) Under variable-power pulsed light, photocurrent transients take the form of exponential growth/decay curves (Fig. 5a). The associated rise and fall times (taken as the times for the photocurrent to cover 50% of its full-range transition) are plotted in Fig. 5b, their average ranging from ~ 45 to ~ 175 ms. In principle, the charge–discharge of the photodetector capacitance may pose a limitation on its speed. To evaluate this effect, we measured the capacitance of Ag_2_BiI_5_ photodetectors (Fig. 5c). We found that the capacitance was a strong function of frequency, as often reported in the literature of related optoelectronic materials [35, 36]. As discussed in Note S5, the frequency range of interest for a comparison with the observed photodetector transients (Fig. 5a) was 1–5 Hz. When the capacitance values at such frequencies are considered, the RC time constant in the worst-case scenario was 400 μs (Note S5). This value is significantly smaller than any of the response times observed experimentally. Therefore, we concluded that the observed photocurrent transients were not affected by the charge/discharge of the photodetector capacitance.

With RC effects ruled out, the photodetector transient response necessarily relates to either carrier transit and/or to trapping dynamics. In this respect, the reduction in response time with optical power is particularly revealing. In principle, a boost in carrier mobility with optical power could justify the observed dependence. However, this would be at variance with the $I_{\text{ph}} - P_{\text{opt}}$ linearity (Note S6). In addition, our Hall mobility characterization (Fig. 5d and Table S1) allowed us to obtain an order-of-magnitude estimate of the carrier transit time $t_{\text{tr}}$ ($t_{\text{tr}} = L /\left( {\mu E} \right)$, where $L$ is the distance that carriers must travel to reach the respective electrodes, and $E$ is the internal electric field). In particular, an upper estimate can be obtained by taking $L$ as the entire photoactive thickness, which generates a $t_{\text{tr}}$ in the range of 1–10 ns. Being many orders of magnitude shorter than the observed response times, this estimate further indicates that charge transport is not expected to limit the speed of the response of our photodetectors.

A reduction in the response time with optical power can be alternatively rationalized in terms of trapping effects. In fact, the inverse dependence of the response time on optical power matches the behavior associated with a one-center model featuring a uniform trap distribution (Fig. 4b), which is consistent with the Rose–Bube theoretical framework (Note S7). It is worth noting that this model is also fully compatible with our earlier hypothesis on monomolecular recombination and with the observed $I_{\text{ph}} - P_{\text{opt}}$ linearity (Note S4). In the presence of traps, the photocurrent response time $\tau_{\text{r}}$ is prolonged in proportion to the ratio between the number of trapped carriers $n_{\text{t}}$ and the number of free carriers $n_{\text{f}}$: $\tau_{\text{r}} \propto n_{\text{t}} /n_{\text{f}}$ [37]. For a uniform trap density with the quasi-Fermi level lying in its midst, the number of traps filled or emptied upon switching the incident light on or off (i.e., the traps within $kT$ from the quasi-Fermi level) is approximately constant, whereas the number of free carriers increases with incident optical power. All of this results in an inverse dependence of $\tau_{\text{r}}$ on $P_{\text{opt}}$. Concurrently, the filling/emptying of these traps has a negligible effect on the carrier lifetime (and, hence, on the photocurrent; see Note S4). Clearly, this is consistent with the observed $I_{\text{ph}} - P_{\text{opt}}$ linearity.

In addition to providing insight into Ag_2_BiI_5_ optoelectronics, the observed photocurrent transients also have technological implications. Indeed, the response time magnitude reveals that the Ag_2_BiI_5_ photodetectors presented here meet the speed requirements for many applications (e.g., environmental monitoring, tracking of biological parameters for diagnostic purposes, and smart homes). Regarding applications requiring shorter response times (e.g., visible light communications, high-speed cameras), our analysis indicates that the passivation of the trap states that limit the response time of Ag_2_BiI_5_ photodetectors constitutes a worthwhile goal for future research that seeks to expand their application scope.

## Conclusion

This study provided the first assessment of the photodetection capabilities of Ag_2_BiI_5_, an emerging perovskite-inspired, lead-free, solution-processible semiconductor. By exploring different solution-based deposition methods and device architectures, this study successfully capitalized on the optoelectronic properties of Ag_2_BiI_5_ to deliver self-powered visible light photodetection. Using a photoactive layer in which Ag_2_BiI_5_ was predominantly interspersed within a mesoporous titania matrix, we demonstrated (in self-powered mode) a near-constant > 100 mA W^−1^ responsivity through the visible range, a near-infrared rejection ratio of > 250, and a long-wavelength responsivity onset that well matched standard colorimetric functions. In addition, Ag_2_BiI_5_ photodetectors exhibited a photocurrent response linear with optical power (which is an essential functional requirement for photodetection) over more than 5 orders of magnitude. This reveals that Ag_2_BiI_5_ offers an appealing platform for NIR-blind, visible-light/color detection, which can also potentially address the broad range of applications requiring photometric capability and/or photodetection under low-light conditions.

In addition to the aforementioned technological implications, we used our experimental findings to gain insight into Ag_2_BiI_5_ optoelectronics. We found that the quantum efficiency trends could be rationalized in terms of differences in carrier collection distance when considering the limited magnitude of the carrier drift length in self-powered mode. In addition, we showed that the state-of-the-art photocurrent-power linearity and the transient behavior of Ag_2_BiI_5_ (HIA) were consistent with a one-center model featuring a uniform trap distribution. In perspective, these results highlight that a major priority in Ag_2_BiI_5_ optoelectronic research lies in the identification and passivation of defects acting as recombination centers and traps.

By demonstrating the photodetection capabilities of Ag_2_BiI_5_, and by providing insight into its underlying optoelectronic properties, this study constitutes an important step in developing a low-toxicity solution-processible platform for NIR-blind visible light photodetectors for emerging off-the-grid applications.

## Electronic supplementary material

Below is the link to the electronic supplementary material.

Supplementary material 1 (PDF 1114 kb)
