# Supplementary material for: Perovskite-Inspired Lead-Free Ag2BiI5 for Self-Powered NIR-Blind Visible Light Photodetection
Source: Nanomicro Lett. 2020 Jan 20;12:27. doi: 10.1007/s40820-020-0371-0 (PMC7770846; doi:10.1007/s40820-020-0371-0)
Supplement: Supplementary file 1 — Supplementary material 1 (PDF 1114 kb) [file 40820_2020_371_MOESM1_ESM.pdf]

Supporting Information For

Perovskite-Inspired Lead-Free Ag_2BiI_5 for Self-Powered NIR-Blind Visible Light Photodetection

Vincenzo Pecunia[1, #, *], Yue Yuan[1, #], Jing Zhao[1], Kai Xia[1], Yan Wang[1], Steffen Duhm[1], Luis Portilla[1], Fengzhu Li[1]

[1]Institute of Functional Nano & Soft Materials (FUNSOM), Jiangsu Key Laboratory for Carbon-Based Functional Materials & Devices, Soochow University, 199 Ren'ai Road, Suzhou, 215123 Jiangsu, People's Republic of China

#Vincenzo Pecunia and Yue Yuan contributed equally to this work

*Corresponding author. E-mail: vp293@suda.edu.cn (Vincenzo Pecunia)

Supplementary Figures

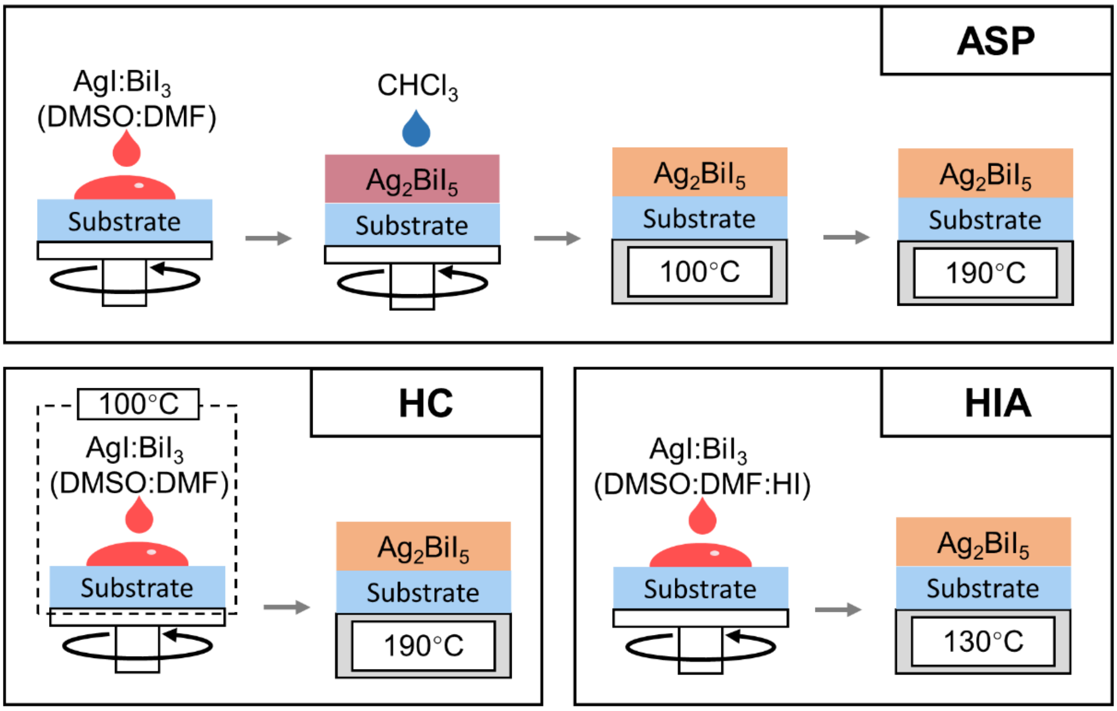

Fig. S1 Schematic depiction of the different Ag_2BiI_5 deposition methods considered in this study: antisolvent processing (ASP), hot coating (HC), and hydroiodic acid additive (HIA)

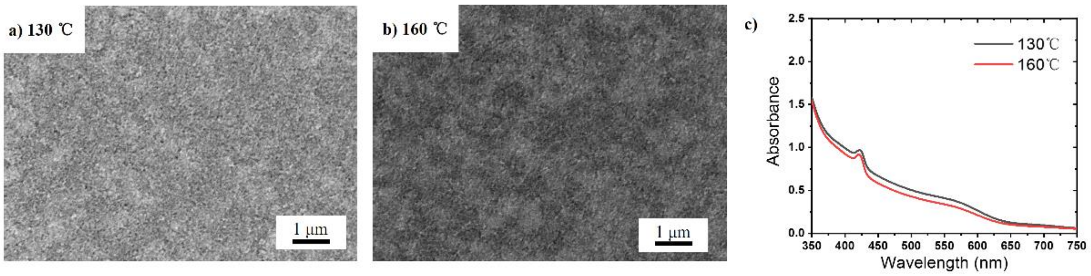

Fig. S2 Impact of annealing temperature on HIA-processed films: **a**) 130 °C; **b**) 160 °C. **c**) Corresponding absorbance. No significant change from SEM and absorbance characterization is detected

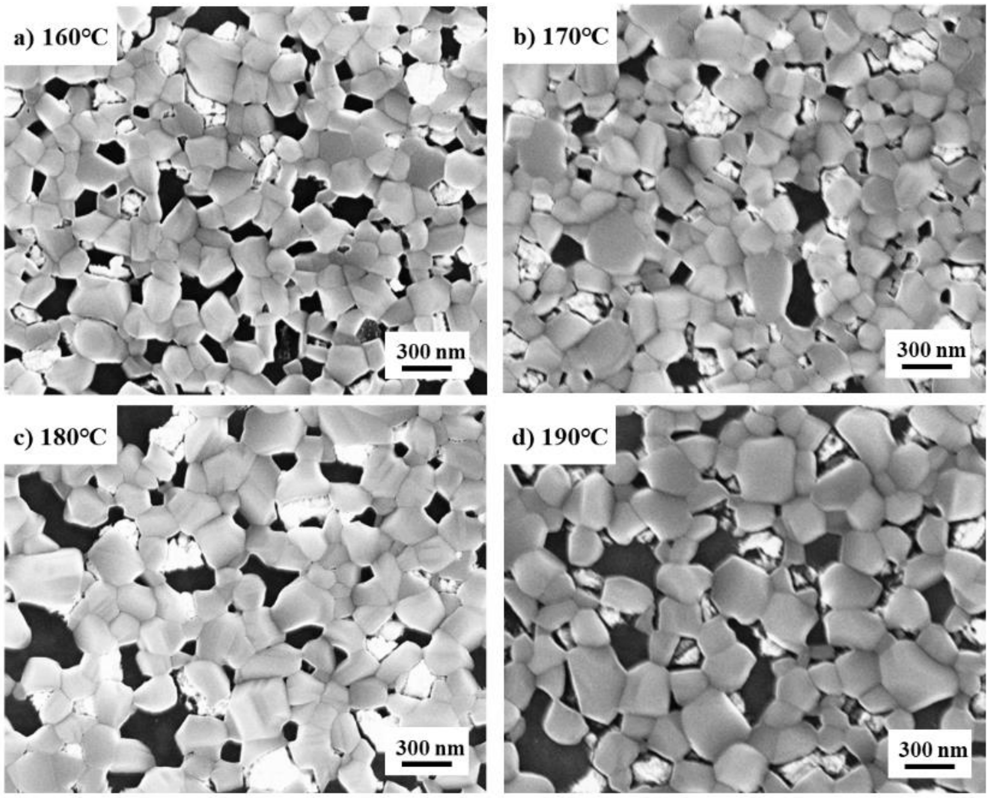

Fig. S3 Impact of annealing temperature on ASP-processed films (without pre-annealing): **a**) 160 °C; **b**) 170 °C; **c**) 180 °C; **d**) 190 °C. An improvement in grain size with annealing temperature is observed, the largest grain size being obtained with an annealing temperature of 190 °C. In order to suppress pinhole formation, an additional pre-annealing step at 100 °C—followed by annealing at 190 °C—was introduced, leading to the optimized ASP films shown in Fig. 1a.

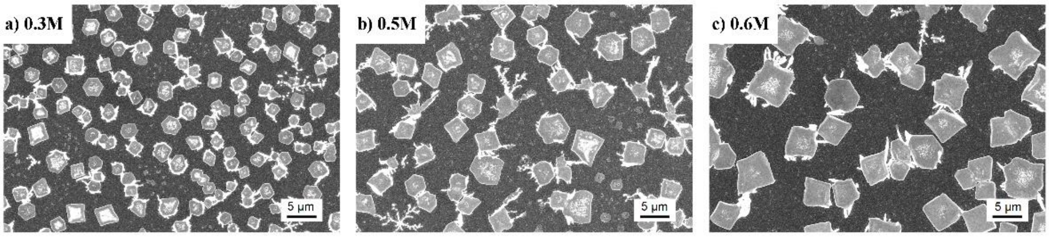

Fig. S4 SEM images of Ag_2BiI_5 (HIA) deposited (on c-TiO_2) with increasing molarity of the precursor solution: **a**) 0.3 M; **b**) 0.5 M; and **c**) 0.6 M. Concentrations higher than 0.8 M could not be considered due to solution saturation (at room temperature). In all cases, disconnected crystallites (whose size increases with concentration) are left on the substrate, i.e., a film is not formed. This finding is independent of the spin-coating speed and the amount of solution dispensed on the substrate.

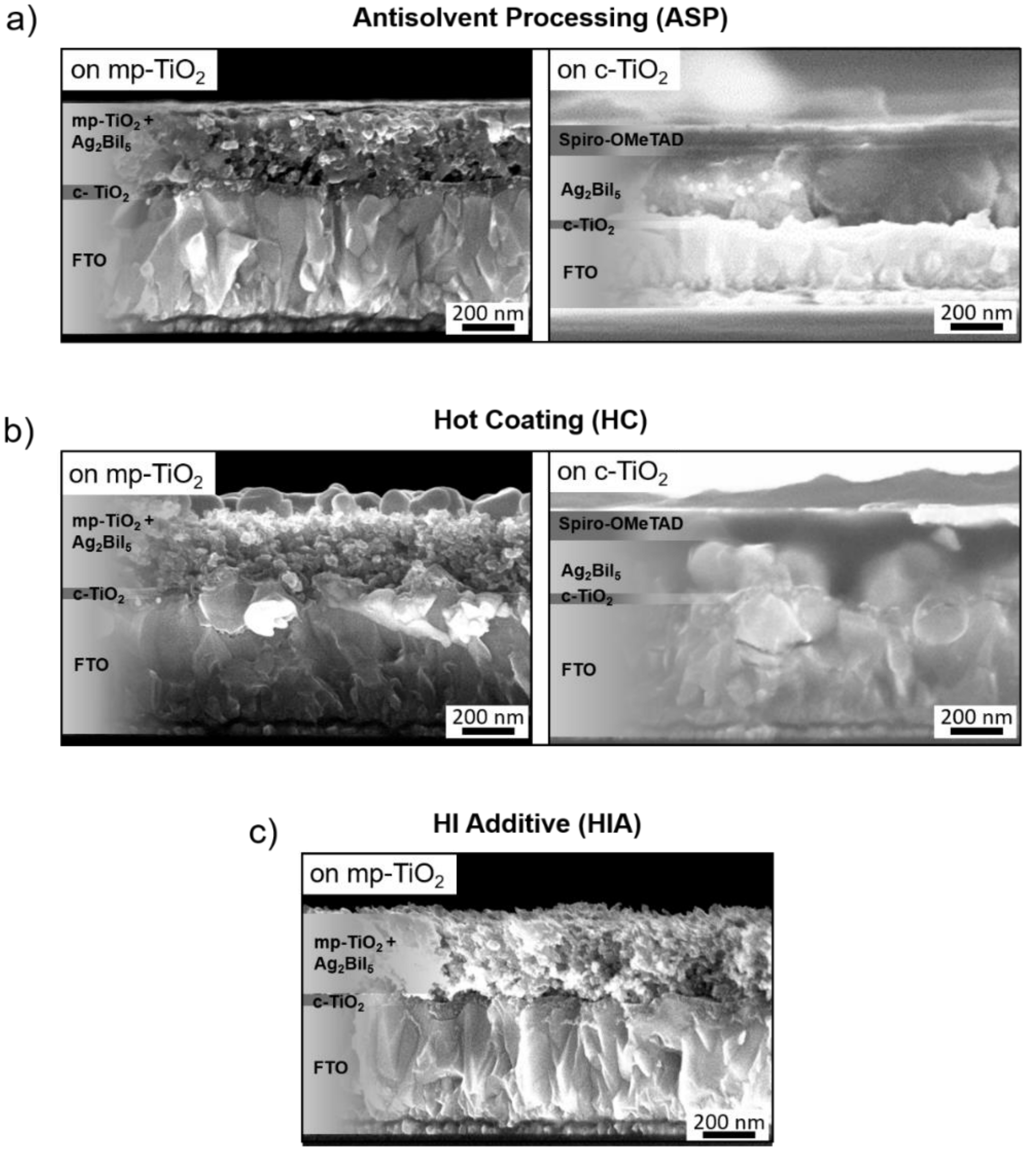

Fig. S5 Cross-sectional SEM images of the different sample types studied in this work. **a**) ASP samples; **b**) HC samples; **c**) HIA samples. All Ag_2BiI_5 layers were deposited on Glass|FTO|TiO_2.

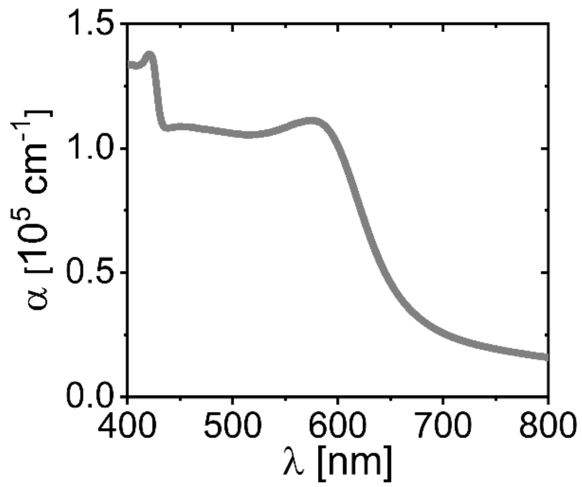

Fig. S6 Absorption coefficient of Ag_2BiI_5 extracted from UV-Vis absorption spectrometry of compact HC films. In-band values are in excess of $1{\cdot}10^5$ cm^{-1}. The apparent non-zero absorption coefficient at long wavelengths is expected to result from film roughness [S1].

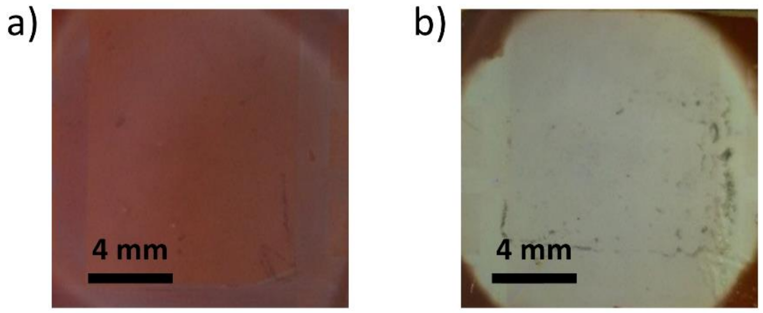

Fig. S7 Effect of the spin coating of a solution of Spiro-OMeTAD comprising tert-butylpyridine (tBP) and lithium bis(trifluoromethylsulfonyl)-imide (LiTFSI) on a Ag_2BiI_5 layer. **a**) Ag_2BiI_5 layer before spin coating a Spiro-OMeTAD:tBP-LiTFSI solution. **b**) Sample after spin coating a Spiro-OMeTAD:tBP-LiTFSI solution.

Note S1 – Specific Detectivity of Ag_2BiI_5 Photodetectors

While the primary aim of the present work concerns the assessment of the photoconversion capabilities of Ag_2BiI_5 photodetectors, here we briefly present on their noise and detectivity. This is in consideration of future research avenues aiming at the realization of the full potential of Ag_2BiI_5 photodetectors for NIR-blind visible light photodetection.

The specific detectivity $D^*(\lambda)$ of a photodetector constitutes an aggregate measure of how its responsivity $R(\lambda)$ compares to its photocurrent noise: $D^*(\lambda) := R(\lambda) \cdot \sqrt{A_{ph} \cdot \Delta f} / i_n$ [S2]. Here, A_{ph} is the photodetector input area, and Δf is the frequency bandwidth over which the photocurrent noise i_n (root-mean-square value) is acquired.

To the end of estimating the specific detectivity of our photodetectors, we consider the following contributions to the photocurrent noise: a) shot noise, which manifests a

power spectral density $S_{shot} = 2qI_D$, where q is the elementary charge, and I_D is the photodetector dark current; b) Johnson-Nyquist noise, which exhibits a power spectral density $S_{JN} = 4kT/R_{sh}$, where k is the Boltzmann constant, T is the absolute temperature, and R_{sh} is the photodetector shunt resistance. Considering that Ag_2BiI_5 (HIA) photodetectors lead to the highest photoconversion efficiency, we narrow down the discussion to this type of photodetectors in the following. The dark current and shunt resistance values of such photodetectors were obtained from their current-voltage (*I*-*V*) characteristics measured around 0 V (applied bias voltage). The average value of the dark current at 0 V across different devices is 0.9 nA. The shunt resistance was derived from the slope of the I-V characteristics around 0 V, and its average value across different devices amounts to 662 kΩ. This leads to the following estimate of the noise contributions in Ag_2BiI_5 (HIA) photodetectors: $S_{shot} = 2.9 \cdot 10^{-28}\ A^2Hz^{-1}$ and $S_{JN} = 2.5 \cdot 10^{-26}\ A^2Hz^{-1}$. This indicates that the Johnson-Nyquist contribution is dominant for an applied bias of 0 V. In fact, this is expected, as the shot noise contribution is reduced to a minimum in self-powered operation, while the Johnson-Nyquist contribution is independent of the bias point. In spite of the general dominance of the Johnson-Nyquist noise at small applied bias, it is noteworthy that the specific detectivity estimates provided, e.g., in the perovskite photodetector literature typically neglect this contribution, likely leading to a significant misrepresentation of the noise performance and the specific detectivity of the corresponding photodetectors.

The resulting specific detectivity of our Ag_2BiI_5 photodetectors is shown **Fig. S8**. It reaches $\approx\ 2 \times 10^{11}\, Jones$, while following the same spectral dependence of the responsivity (*cf.* Fig. 3c). It is noteworthy that, had we only considered the shot noise contribution (as typically seen, e.g., in the perovskite photodetector literature), the estimated peak specific detectivity would have reached $\approx\ 2 \times 10^{12}\, Jones$ (*i.e.*, it would have been an order of magnitude higher). This points to the need of considering the Johnson-Nyquist noise contribution to avoid misrepresenting the noise performance and specific detectivity of a photodetector—especially at low applied bias.

A detectivity in the $10^{11}\, Jones$ range indicates that Ag_2BiI_5 photodetectors have good sensitivity in low-light conditions. Additionally, such a detectivity represents a good foundation for future optimization work. Indeed, while lower than that of long-established Silicon photodetector technology ($\approx\ 4 \times 10^{12}\, Jones$) [S3, S4], the detectivity of Ag_2BiI_5 photodetectors reported here does not result from a dedicated optimization effort, *i.e.*, it does constitute the ultimate detectivity limit of Ag_2BiI_5. Therefore, it can be envisaged that higher detectivity could be achieved by optimizing the photoactive layer and the overall device architecture, for instance, by devising measures to increase the shunt resistance while boosting the photoconversion efficiency.

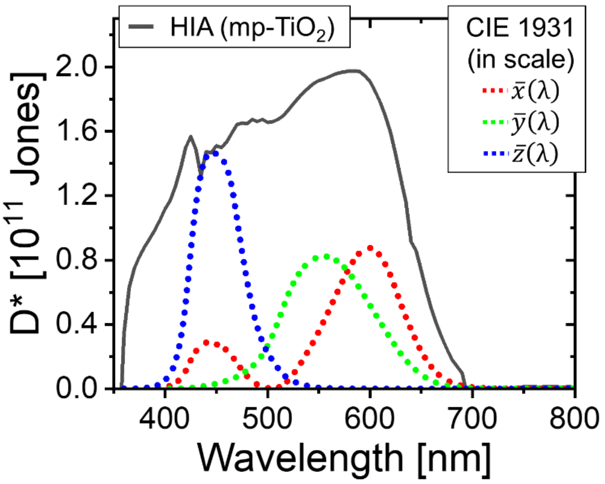

Fig. S8 Estimated specific detectivity of HIA Ag_2BiI_5 photodetectors. The estimate is based on the noise analysis detailed in Note S1. The CIE 1931 functions [S5] (in scale) are also shown so as to illustrate their spectral match with the specific detectivity of HIA Ag_2BiI_5 photodetectors, as relevant to colorimetric and color imaging applications.

Note S2 – Internal Quantum Efficiency of Ag_2BiI_5 Photodetectors

The internal quantum efficiency (IQE) of all photodetector types (**Fig. S9**) was determined from the corresponding experimental EQE. To this end, the absorbance $A(\lambda)$ of each of the device stacks was measured so as to firstly derive the associated absorption efficiency (*i.e.*, the ratio between the number of absorbed photons and the number of incident photons): $\eta_{abs}(\lambda) = \left(1 - 10^{-A(\lambda)}\right)$. Finally, the IQE was determined as: $IQE(\lambda) = EQE(\lambda)/\eta_{abs}(\lambda)$. The resulting IQE spectra closely match the corresponding EQE spectra, as expected in consideration of the high absorption efficiencies of all Ag_2BiI_5 layers.

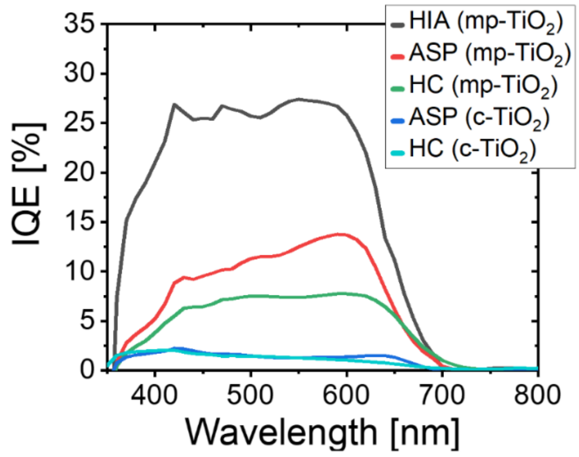

Fig. S9 Calculated IQE as a function of wavelength for all explored device configurations

Note S3 –Linear Dynamic Range of Ag_2BiI_5 Photodetectors

The photocurrent-optical power relationship shown in Fig. 3d indicates that, while a deviation from linearity occurs at very high optical power, linearity is maintained down to the lowest power values measurable with our apparatus. Considering that the linear dynamic range is defined in relation to the full range over which a photodetector holds a linear behavior, it follows that the linear dynamic range of the Ag_2BiI_5 (HIA) devices is expectedly larger than that inferred from Fig. 3d. In fact, if a photodetector exhibits a broad linear photocurrent-power relationship, it is generally the case that the limit below which linearity is compromised corresponds to having $I_{ph} = i_n$, where i_n is the noise current (root-mean-square value). Indeed, optical power values below this limit (minimum detectable power) would result in a photocurrent indistinguishable from noise, *i.e.*, the total photodetector current would saturate and linearity would no longer hold.

With the aim of assessing the upper limit on the linear dynamic range of Ag_2BiI_5 (HIA) photodetectors, here we consider the implications of their minimum detectable power on their dynamic range. To this end, it is useful to extrapolate the linear dependence of the $I_{ph} - P_{opt}$ dataset, identifying the point at which the photocurrent reaches the noise current estimated in Note S1. As shown in **Fig. S10**, the upper estimate of the linear dynamic range of Ag_2BiI_5 (HIA) photodetectors is 182 dB ($LDR = 20\ \log_{10}(I_{ph,max}/I_{ph,min})$, $I_{ph,max}$ and $I_{ph,min}$ being the upper and lower limits of the linear range). This indicates that the linear $I_{ph} - P_{opt}$ relationship is could potentially span over 9 orders of magnitude. Consequently, Ag_2BiI_5 (HIA) has potential for photodetection in low-light conditions, specifically down to an optical power areal density in the tens of $pW\ cm^{-2}$. Additionally, this implies that future works aiming to unravel the potential of Ag_2BiI_5 (HIA) for sensing in low-light conditions are to probe down to this power range.

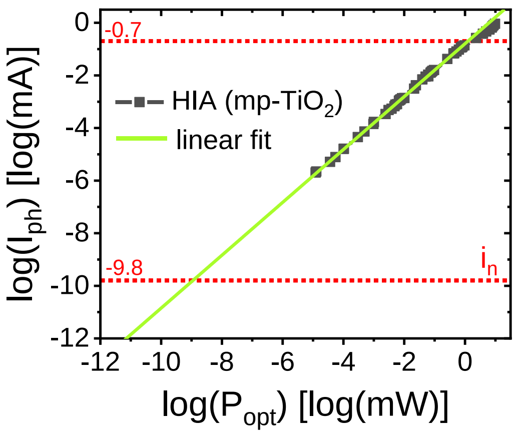

Fig. S10 $I_{ph} - P_{opt}$ relationship in Ag_2BiI_5 (HIA) photodetectors, along with its linear fit. The upper red dotted line denotes the linearity limit observed experimentally at high optical power, while the lower one denotes the minimum detectable photocurrent. This allows us to estimate that the upper limit on the dynamic range of Ag_2BiI_5 (HIA) photodetectors is 182 dB.

Note S4 – Photocurrent vs. Optical Power: One-center Models with Traps

The relationship between the photocurrent (I_{ph}) and the optical power (P_{opt}) in semiconductors has been the subject of extensive research. A range of models have been developed to rationalize experimental observations, fundamental contributions to which have been made by A. Rose and R. H. Bube [S6, S7]. These models rationalize photocurrent data from manifold semiconductor systems [S8–S13], and in fact they also offer a consistent picture of our findings from Ag_2BiI_5. While these models have been covered in detail in the works of R.H. Bube, A. Rose, and A. M. Goodman [S6, S7, S14], here we present a summary of their key aspects so as to offer an immediate context for the discussion on Ag_2BiI_5 photodetectors in the main text.

It has been generally found that I_{ph} manifests a power-law dependence on P_{opt}. Therefore, in terms of the photogeneration rate f (*i.e.*, the number of electron-hole pairs generated per unit volume and per unit time, $f \propto P_{opt}$), the following expression generally holds:

$$I_{ph} = c \cdot f^{\gamma} \quad \text{(S1)}$$

Here, c is a constant, and the exponent γ accounts for any observed non-linearity between the photocurrent and the light intensity.

Experimental findings from manifold semiconductors can be rationalized within the framework of so-called one-center models with traps [S8–S13]. These models account for recombination as occurring via defects that are approximately mono-energetic (*i.e.*, narrowly distributed deep in the forbidden gap) and with single-valued electron and hole capture cross sections (hence the attribute *one-center*). Moreover, these models comprise additional defect levels, which differ from the former insofar as they act as charge carrier traps. The distinction between recombination centers and traps relates to the relative probability that carrier capture is followed by re-emission (as in the case of a trap) or by the capture of a carrier of opposite polarity (as in the case of a recombination center).

In a one-center model without traps, the photocurrent may manifest a square-root dependence on optical power (*i.e.*, $\gamma = 1/2$) at low P_{opt}, and a linear dependence (*i.e.*, $\gamma = 1$) at high P_{opt}. On the contrary, if traps are present, γ values between $1/2$ and 1 become possible. This result can be rationalized in terms of the trap distributions that have been typically found in semiconductors. Such distributions can be classified into two categories (**Fig. S11**): a) traps uniformly distributed in energy (*i.e.*, with a density of states $n_{tE}(E)dE = n_{t0}dE$, n_{t0} being a constant); b) traps distributed exponentially in energy (*i.e.*, with a density of states $n_{tE}(E)dE = n_{exp0}\exp(-|E|/(kT_0))dE$, n_{exp0} being a constant, T_0 a characteristic temperature, $T_0 > T$, and E being referenced to a band edge).

As a working hypothesis for us to present the $I_{ph} - P_{opt}$ trends corresponding to the typical trap distributions within the concise space of this note, we narrow down our focus to the case in which carriers of a given type—*e.g.*, holes—are more readily captured by the recombination centers. Trapping is then relevant only to carriers of the

other type—*i.e.*, electrons. Consequently, the photocurrent closely relates to the steady-state free electron density n_f, which can be expressed as:

$$n_f = f/(\beta_n \cdot p_r) \tag{S2}$$

where β_n is the free-electron capture coefficient of the recombination centers, and p_r is the density of holes captured by the recombination centers. It is important to note that trapping is significant only for optical power values such that the total density of trapped electrons (n_t) is much greater than the density of free electrons (n_f). In view of charge neutrality, this corresponds to having $p_r \cong n_t$. As here we are specifically concerned with the impact of trapping, the discussion that follows refers to this optical power range.

It can be easily seen that a uniform trap distribution results in a $I_{ph} - P_{opt}$ dependence with $\gamma = 1/2$ or $\gamma = 1$. Indicating with E_{fn} the electron quasi-Fermi level, we concisely present here the following three cases (**Fig. S11**a-c): (a) E_{fn} located above the trap distribution; (b) E_{fn} located within of the trap distribution; (c) E_{fn} located below the trap distribution. If E_{fn} is above the trap distribution (case a)), then, according to Eq. S1, the photogeneration rate must be an exponential function of E_{fn} (just like n_f, *cf.* Boltzmann statistics), as concurrently n_t (and p_r) are constant. In other words, $n_f \propto f$, *i.e.*, $\gamma = 1$. Case (b) follows this same trend, as the photogeneration rate must be approximately an exponential function of E_{fn} to accommodate an exponential growth in n_f , considering that p_r concurrently undergoes a rather modest change (linear in E_{fn}). In other words, (b) also gives $n_f \propto f$, *i.e.*, $\gamma = 1$. Finally, if E_{fn} is below the trap distribution (case (c)), then n_t varies exponentially with E_{fn} just like n_f (*cf.* Boltzmann statistics), *i.e.*, $n_t \propto n_f$. As $p_r \cong n_t$ in the optical power range being considered, then Eq. S1 leads to $n_f^2 \propto f$, *i.e.*, $\gamma = 1/2$.

In contrast, in the case of an exponential trap distribution (**Fig. S11**d), γ can take any value between $\gamma = 1/2$ and $\gamma = 1$. Indeed, n_t is now given by the convolution of the exponential density of trap states and a Boltzmann factor, leading to $n_t = kT_0 n_{expo0} \exp\left(-E_{fn}/(kT_0)\right)$. As $p_r \cong n_t$ in the optical power range being considered, then Eq. S2 gives $n_f \propto f^{T_0/(T+T_0)}$, *i.e.*, $1/2 < \gamma < 1$.

The cases we have considered thus far implicitly rely on the assumption that the carrier density is uniform within the volume of the semiconducting layer. If, however, this is not the case due to the formation of a space charge region (*i.e.*, a region with near-immobile charge carriers of one type), then the photocurrent can reach a space-charge-limit regime at high P_{opt} due to electrostatic limitations. Within this regime, it is found that $I_{ph} \propto f^{3/4}$, *i.e.*, $\gamma = 3/4$ [S14, S15].

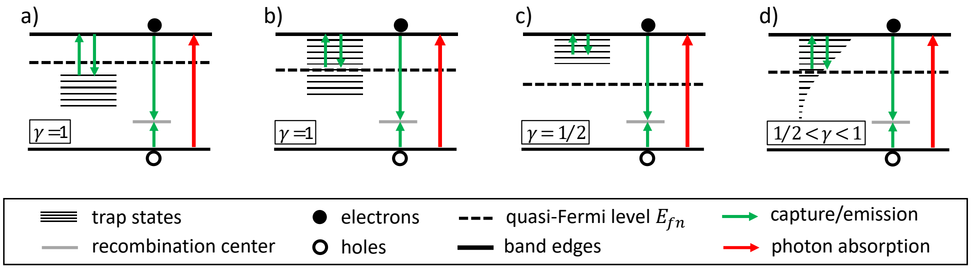

Fig. S11 Schematic depiction of one-center models with traps. Quasi-Fermi level **a)** above, **b)** within, and **c)** below a uniform trap distribution. d) Quasi-Fermi level within an exponential trap distribution. The resulting $I_{ph} - P_{opt}$ relationship is synthetically indicated through the corresponding γ (value or range).

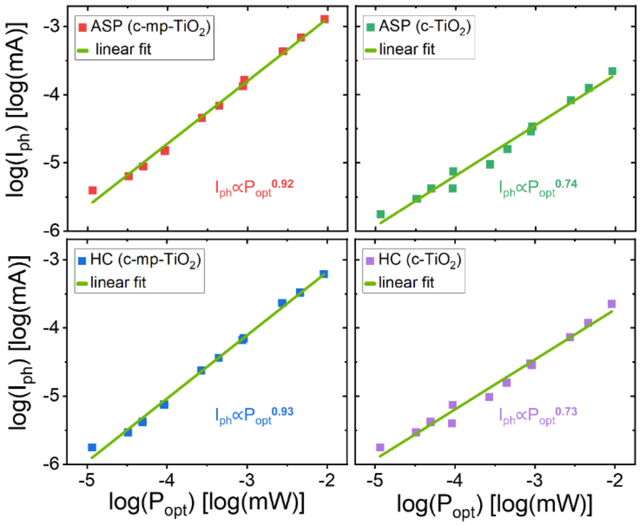

Fig. S12 $I_{ph} - P_{opt}$ characteristics of ASP- and HC-processed Ag_2BiI_5 photodetectors, along with power-law fits, i.e., fits of the type $I_{ph} \propto P_{opt}^{\gamma}$. As $\gamma < 1$ in all cases, the photodetectors do not manifest a linear behavior, contrary to the case of HIA-processed Ag_2BiI_5 photodetectors (see main text for more details and a discussion of the implications).

Note S5 – Analysis of RC Charge-Discharge in Ag_2BiI_5 (HIA) Photodetectors

The charge/discharge of the capacitance associated with a photodetector may pose a limitation on its speed of response. The timescale relevant to such charge/discharge is quantified by the RC time constant τ_{RC}, defined as the product between the equivalent capacitance and the equivalent resistance between the terminals of a photodetector. As a means of assessing the impact of the RC time constant on the photocurrent transients discussed in the main text, here we provide an analysis of the magnitude of τ_{RC}.

When measuring the photocurrent at 0 V (applied voltage bias) with a transimpedance amplifier (as in our photocurrent transient measurements), the RC time constant is approximately equal to the product of the photodetector capacitance and the amplifier input resistance [S2]. Indeed, the shunt resistance of our photodetectors (662 kΩ at 0

V) is orders of magnitude larger than the input resistance of the transimpedance amplifier (50 – 10 kΩ) employed, hence the latter dominates the equivalent resistance. On the contrary, the equivalent capacitance is primarily determined by the photodetector capacitance. The latter was independently measured, and its magnitude around an applied bias of 0 V is shown as a function of frequency in Fig. 5c. As the capacitance is a strong function of frequency, the frequency range corresponding to the observed photocurrent transients has to be identified in order to determine the relevant capacitance values. Considering that the measured rise and fall times are in the 20 – 250 ms range (Fig. 5b), the corresponding frequency range relevant to the determination of the photodetector capacitance can be calculated from the expression $\ln 2/(2\pi t_{r/f})$, where $t_{r/f}$ is the rise/fall time. Indeed, this relation links the timescale of an exponential transient to its frequency-domain equivalent. The resulting frequency range is 1 – 5 Hz. The photodetector capacitance values over this range (along with the amplifier input resistance) allow us to estimate the RC time constant in the worst-case scenario (*i.e.*, longest possible time constant): $max(\tau_{RC}) \cong 400\ \mu s$. As this value is significantly smaller than any of the response times observed experimentally (Fig. 5b), we conclude that the observed photocurrent transients are not affected by the speed limitation posed by the charge/discharge of the photodetector capacitance.

Note S6 – Response Time vs. Optical Power: Effect of Transit Time

The observed reduction of the response time of Ag_2BiI_5 (HIA) photodetectors with optical power (Fig. 5b) could in principle result from a concurrent reduction of the carrier transit time t_{tr}. In fact, as found in some semiconductor systems, it can be envisaged that a boost in carrier mobility μ may ensue light absorption. In such a case, an increase in mobility with optical power is expected. In turn, this would result in a reduction of the photodetector response time, in view of the relationship $t_{tr} = L/(\mu \cdot E)$ (L being the distance that carriers have to travel to reach the respective electrodes, and E the internal electric field).

While offering a possible explanation for the reduction of the response time with optical power, in actuality this model is not compatible with other experimental observations on Ag_2BiI_5 (HIA). In fact, a linear increase of carrier concentration with optical power is inferred from our static optoelectronic characterization of Ag_2BiI_5 (HIA). If one then considers the proportionality between the collection efficiency and carrier mobility ($\eta_C \propto \mu \cdot \tau \cdot E/L$), a rise in mobility with optical power would necessarily dictate a super-linear $I_{ph} - P_{opt}$ characteristic ($I_{ph} \propto P_{opt}^{\gamma}$ with $\gamma > 1$). This being contrary to the observed linearity of the $I_{ph} - P_{opt}$ characteristic (Fig. 3d), it follows that the response time reduction with optical power does not originate from a change in carrier transit time.

Note S7 – Response Time vs. Optical Power: One-center Model with Uniform Trap Distribution

Here we concisely present on the relationship between the photocurrent transient behavior and optical power within a one-center model with a uniform trap distribution (see Note S4 for details on the latter). This serves as a summary of the analysis by A. Rose [S6]—which is consistent with our findings on Ag_2BiI_5 (HIA)—so as to offer an immediate context for the discussion in the main text.

It is well established that the presence of traps results in slower photocurrent transients, in view of the additional time required to fill/empty the traps [S16]. This effect can be generally described in terms of the response time τ_{re}, here defined as the time for the photocurrent to cover a fraction $(1-e^{-1})$ of the full photocurrent variation when illuminated by a light pulse. From this very definition, it follows that τ_{re} corresponds to the time required for a shift of the quasi-Fermi levels by kT away from the initial steady-state values. This is because the carrier densities are proportional to $\exp\left(-\left|E_{fn,fp}\right|/(kT)\right)$ ($E_{fn,fp}$ denoting the electron/hole quasi-Fermi level referred to the conduction/valence band edge).

The one-center models with traps constitute a powerful tool to rationalize the impact of trapping on the response time τ_{re}. Within the general assumptions presented in Note S4, this effect can be expressed as:

$$\tau_{re}=\tau_f\cdot\left(1+\frac{n_{t,kT}}{n_f}\right) \tag{S3}$$

where τ_f is the free electron lifetime, and $n_{t,kT}$ is the trap density corresponding to the slice of width kT swept by the quasi-Fermi level from the start of the photocurrent transition [S17].

We now turn to the implications of Eq. S3 in the presence of a uniform trap distribution ($n_{tE}(E)dE=n_{t0}dE$), assuming that the quasi-Fermi level is in its midst at the start of the transition. The specifics of this trap distribution result in $n_{t,kT}=n_{t0}kT$. Additionally, the case considered here yields $n_f=f\cdot\tau_f$ with $\tau_f\cong constant$ (see Note S4). Therefore:

$$\tau_{re}=\tau_f\cdot\left(1+\frac{n_{t0}kT}{f\tau_f}\right)\cong\frac{n_{t0}kT}{f} \tag{S4}$$

where the final approximation holds in the P_{opt} range where trapping is significant ($n_t\gg n_f$) [S16]. In summary, having the quasi-Fermi level within a uniform trap distribution not only leads to a linear $I_{ph}-P_{opt}$ relationship (see Note S4), but also gives an inverse dependence of the response time on optical power. To a good approximation, this corresponds to our experimental findings on Ag_2BiI_5 (HIA).

Table S1 Hall effect parameters from HC and ASP Ag_2BiI_5 samples. Experiments relied on the van der Pauw configuration. The applied a.c. magnetic field was 0.42291 T, with a modulation frequency of 100 mHz

**ASP**					**HC**				
**Current (nA)**	**Resistivity [kΩ·cm]**	**μ_H [cm²V⁻¹s⁻¹]**	**Carrier Conc. [10¹⁵/cm³]**	**Hall Voltage [μV]**	**Current (pA)**	**Resistivity [MΩ·cm]**	**μ_H [10⁻¹cm²V⁻¹s⁻¹]**	**Carrier Conc. [10¹²/cm³]**	**Hall Voltage [μV]**
10	2.99	1.93	1.07	244	40	15.4	4.88	1.27	271
12	2.92	1.75	1.21	261	50	15.6	3.34	1.86	225
14	3.02	1.61	1.27	290	60	16.0	8.35	0.74	680
16	2.90	1.41	1.52	278	70	15.9	5.66	1.10	541
18	3.04	1.09	1.42	332	80	16.7	6.02	1.03	662

Supplementary References

[S1] M. Shirayama, H. Kadowaki, T. Miyadera, T. Sugita, M. Tamakoshi et al., Optical transitions in hybrid perovskite solar cells: ellipsometry, density functional theory, and quantum efficiency analyses for $CH_3NH_3PbI_3$. Phys. Rev. Appl. **2016**, 5, 14012. https://doi.org/10.1103/PhysRevApplied.5.014012

[S2] J. Liu, *Photonic Devices* (Cambridge University Press, Cambridge, **2005)**, pp. 926–1017.

[S3] G. Konstantatos, J. Clifford, L. Levina, E.H. Sargent, Sensitive solution-processed visible-wavelength photodetectors. Nat. Photonics **2007**, 1, 531. https://doi.org/10.1038/nphoton.2007.147

[S4] X. Gong, M. Tong, Y. Xia, W. Cai, J.S. Moon et al., High-detectivity polymer photodetectors with spectral response from 300 nm to 1450 nm. Science **2009**, 325, 1665. https://doi.org/10.1126/science.1176706

[S5] T. Smith, J. Guild, The C.I.E. colorimetric standards and their use. Trans. Opt. Soc. **1931**, 33, 73. https://doi.org/10.1088/1475-4878/33/3/301

[S6] A. Rose, *Concepts in Photoconductivity and Allied Problems* (John Wiley & Sons, New York, NY, USA, 1963), pp. 11–68.

[S7] R. H. Bube, *Photoelectronic Properties of Semiconductors* (Cambridge University Press, Cambridge, UK, 1992), pp. 45–59.

[S8] G. Marcano, A.R. Zanatta, I. Chambouleyron, Photoconductivity of intrinsic and nitrogen-doped hydrogenated amorphous germanium thin films. J. Appl. Phys. **1994**, 75, 4662. https://doi.org/10.1063/1.355918

[S9] G. Cunningham, D. Hanlon, N. McEvoy, G.S. Duesberg, J.N. Coleman, Large variations in both dark- and photoconductivity in nanosheet networks as nanomaterial is varied from MoS_2 to WTe_2. Nanoscale **2015**, 7, 198. https://doi.org/10.1039/C4NR04951A

[S10] C.R. Wronski, R.E. Daniel, Photoconductivity, trapping, and recombination in discharge-produced, hydrogenated amorphous silicon. Phys. Rev. B **1981**, 23, 794. https://doi.org/10.1103/PhysRevB.23.794

[S11] S. Ghosh, A. Winchester, B. Muchharla, M. Wasala, S. Feng et al., Ultrafast intrinsic photoresponse and direct evidence of sub-gap states in liquid phase exfoliated MoS_2thin films. Sci. Rep. **2015**, 5, 11272. https://doi.org/10.1038/srep11272

[S12] O. Lopez-Sanchez, D. Lembke, M. Kayci, A. Radenovic, A. Kis, Ultrasensitive photodetectors based on monolayer MoS_2. Nat. Nanotechnol. **2013**, 8, 497. https://doi.org/10.1038/nnano.2013.100

[S13] R.S. Crandall, in *Semiconductors and Semimetals - Hydrogenated Amorphous Silicon - Optical Properties*, ed. By J. J. Pankove (Academic Press Inc., London, UK, 1984), pp. 245–298.

[S14] A.M. Goodman, A. Rose, Double extraction of uniformly generated electron‐hole pairs from insulators with noninjecting contacts. J. Appl. Phys. **1971**, 42, 2823. https://doi.org/10.1063/1.1660633

[S15] V.D. Mihailetchi, J. Wildeman, P.W.M. Blom, Space-Charge Limited Photocurrent. Phys. Rev. Lett. **2005**, 94, 126602. https://doi.org/10.1103/PhysRevLett.94.126602

[S16] R.H. Bube, *Photoconductivity of Solids* (John Wiley & Sons, Inc., New York, NY, USA, 1960), pp. 56–87.

[S17] A. Rose, *Concepts in Photoconductivity and Allied Problems* (John Wiley & Sons, New York, NY, USA, 1963), p. 20.
